# Supplementary material for: Complexome profiling of the Chlamydomonas psb28 mutant reveals TEF5 as an early PSII assembly factor
Source: Plant Cell. 2025 Mar 17;37(6):koaf055. doi: 10.1093/plcell/koaf055 (PMC12164590; doi:10.1093/plcell/koaf055)
Supplement: koaf055_Supplementary_Data [file koaf055_supplementary_data.zip › psb28_interactive.zip!/plots/intensity/qualityPlots/peptides/RPL35_Cre14.g617900.t1.2.html]

### Cre14.g617900.t1.2

Trivial name: RPL35  
SeqCoverage [median]: 17.70  
Mass: 14.86 kDa  
  
 

| Sequence | MissedCleavage | Mass [Da] | wtIntensity | muIntensity | Score |
| --- | --- | --- | --- | --- | --- |
| LGSLKGELQQLR | 1 | 1340.78 | 563.09 | 2258.51 | 98.90 |
| VTGGAPNKLSK | 1 | 1070.61 | 579.33 | 578.39 | 51.84 |
|  |  |  | 1142.4 | 2836.9 |  |
